# Mismatch between the eye and the optic lobe in the giant squid

**DOI:** 10.1098/rsos.170289

**Published:** 2017-07-19

**Authors:** Yung-Chieh Liu, Tsung-Han Liu, Chun-Chieh Yu, Chia-Hao Su, Chuan-Chin Chiao

**Affiliations:** 1 Institute of Systems Neuroscience, National Tsing Hua University, Hsinchu, Taiwan, Republic of China; 2 Department of Life Science, National Tsing Hua University, Hsinchu, Taiwan, Republic of China; 3 Institute of Molecular Medicine, National Tsing Hua University, Hsinchu, Taiwan, Republic of China; 4 Institute for Translational Research in Biomedicine, Kaohsiung Chang Gung Memorial Hospital, Kaohsiung, Taiwan, Republic of China

**Keywords:** *Architeuthis*, cortex of optic lobe, medulla of optic lobe, magnetic resonance imaging, cephalopods

## Abstract

Giant squids (*Architeuthis*) are a legendary species among the cephalopods. They live in the deep sea and are well known for their enormous body and giant eyes. It has been suggested that their giant eyes are not adapted for the detection of either mates or prey at distance, but rather are best suited for monitoring very large predators, such as sperm whales, at distances exceeding 120 m and at a depth below 600 m (Nilsson *et al.* 2012 *Curr. Biol.* **22**, 683–688. (doi:10.1016/j.cub.2012.02.031)). However, it is not clear how the brain of giant squids processes visual information. In this study, the optic lobe of a giant squid (*Architeuthis dux*, male, mantle length 89 cm), which was caught by local fishermen off the northeastern coast of Taiwan, was scanned using high-resolution magnetic resonance imaging in order to examine its internal structure. It was evident that the volume ratio of the optic lobe to the eye in the giant squid is much smaller than that in the oval squid (*Sepioteuthis lessoniana*) and the cuttlefish (*Sepia pharaonis*). Furthermore, the cell density in the cortex of the optic lobe is significantly higher in the giant squid than in oval squids and cuttlefish, with the relative thickness of the cortex being much larger in *Architeuthis* optic lobe than in cuttlefish. This indicates that the relative size of the medulla of the optic lobe in the giant squid is disproportionally smaller compared with these two cephalopod species. This morphological study of the giant squid brain, though limited only to the optic lobe, provides the first evidence to support that the optic lobe cortex, the visual information processing area in cephalopods, is well developed in the giant squid. In comparison, the optic lobe medulla, the visuomotor integration centre in cephalopods, is much less developed in the giant squid than other species. This finding suggests that, despite the giant eye and a full-fledged cortex within the optic lobe, the brain of giant squids has not evolved proportionally in terms of performing complex tasks compared with shallow-water cephalopod species.

## 1. Introduction

Giant squids (*Architeuthis dux*) are the second largest cephalopods in the ocean, and their size is only exceeded by the colossal squid (*Mesonychoteuthis hamiltoni*). They can achieve 200 cm in mantle length [1]. It has been reported that giant squids live in the depth range of 300–1000 m [1–3], where the visual environment is extremely dim and almost without sunlight even during daytime [4]. Instead, the main light source at this depth is the bioluminescence from other organisms, such as the photophores on fish and other cephalopods [5,6]. Living in such a low light environment, giant squids have needed to develop a unique visual system that allows them effectively to find prey, predators and mates [7–11].

**Figure 1.**
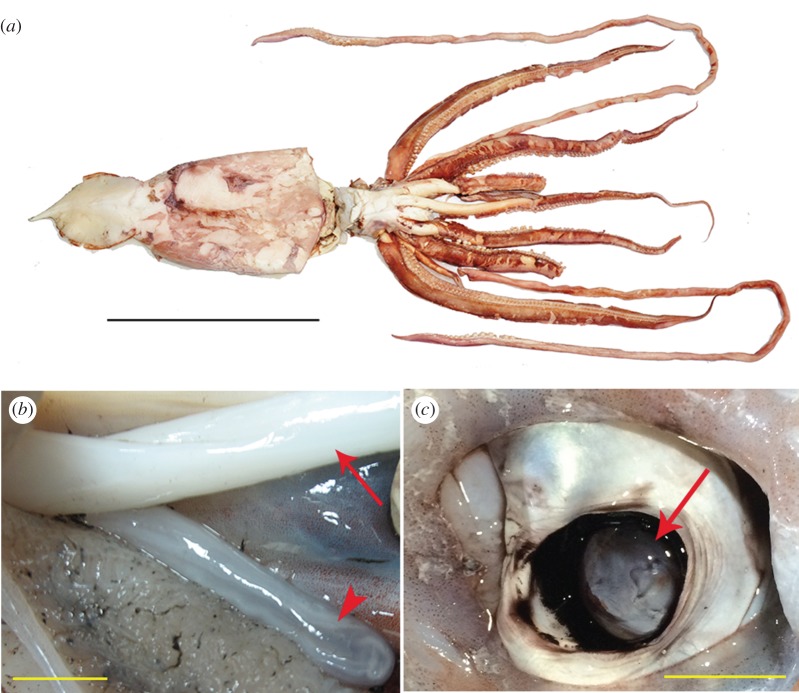
The giant squid specimen. (*a*) The giant squid after fixation. Mantle length, 89 cm when measured freshly but 70 cm after fixation and dehydration; full length, approximately 4 m; scale bar, 50 cm. (*b*) A mature sperm mass (arrowhead) was evident beneath the penis (arrow). Scale bar, 1 cm. (*c*) The giant eye. The eyeball has dropped into the eye socket, thus only the lens (arrow) is visible. Scale bar, 5 cm.

In addition to their large body, giant squids are also known for their enormous eyeballs [4,12]. A previous analysis of the *Architeuthis* eye suggested that the large diameters of the pupil and eye provide giant squids with a special advantage when detecting predators at far distance [13]. The retinal image from the eye in squid is sent to the optic lobe, a major brain area just behind the eyeball, where it undergoes visual information processing [14]. The optic lobe is a complex neural structure that is made up of the outer cortex, also called the deep retina [15], which contains visual analysing systems that process the input from the eye, and the central medulla, which is a higher motor centre responsible for controlling, for example, dynamic body patterns [14,16–18]. Despite there being significant knowledge of the giant eye in *Architeuthis* [13], the morphology and function of the optic lobe in giant squids has not been studied systematically. In this study, a rare sample of the optic lobe from a giant squid (*A. dux*, male, mantle length 89 cm; figure 1) was scanned using high-resolution magnetic resonance imaging (MRI) in order to visualize its general morphology and internal structure. The neural organization in the optic lobe was then quantified and compared with other cephalopod species.

## 2. Material and methods

### 2.1. Animals

Three different species of cephalopods, giant squid, *A. dux* (mantle length 89 cm; figure 1*a*), oval squid, *Sepioteuthis lessoniana* (mantle length 23 cm) and pharaoh cuttlefish, *Sepia pharaonis* (mantle length 16 cm), were captured off the northeastern coast of Taiwan. The giant squid was caught accidentally by local fishermen trawling for mullet (*Mugil cephalus*) near Wushi Port, Yilan, Taiwan. The specimen was identified as *A. dux* [19] based on its general morphology. Although it was captured in shallow water at depth about 18–20 m, presumably while following a shoal of mullets, giant squids usually live much deeper than this. It was noted that this giant squid was a sexually mature adult male with a fully developed sperm mass (figure 1*b*). It was relatively small in size (mantle length 89 cm) for this species, the minimum size for a sexually mature giant squid being recorded to be 17.9 cm mantle length [20].

### 2.2. Tissue preparation

The giant squid was transported to the laboratory at low temperature (less than 4°C) by storing it on ice for approximately 6 h after its capture. The specimen was then dissected at reduced temperature (less than 15°C) over 4 h and then fixed using 10% formalin. The formalin was replaced with 70% ethanol after 10 days and is now preserved permanently in the National Museum of Natural Science, Taichung, Taiwan. The left optic lobe was isolated carefully for MRI scanning. For comparison, the optic lobes of oval squid and a cuttlefish were also obtained. A specimen of each of these two species was anaesthetized using 100 mM magnesium chloride. Once they had ceased to move and their skins had turned pale, the optic lobes were isolated and fixed with 10% formalin for 3 days, which was then replaced with 70% ethanol. The left optic lobes of the oval squid and the cuttlefish were used for the MRI scans.

### 2.3. Magnetic resonance imaging scanning and three-dimensional reconstruction

The MRI scanning of the optic lobes from the three species of cephalopods was carried out at the Kaohsiung Chang Gung Memorial Hospital using a 9.4 T, Bruker BioSpec 94/20 USR MRI machine. Before scanning, the oval squid and cuttlefish samples were embedded in agar containing ferric ions to reduce background noise. In the case of the giant squid sample, it was held steady in a 50 ml centrifuge tube filled with 70% ethanol. The optic lobes were imaged at high resolution using a TurboRARE-3D-torun sequence (TR/TE = 3000/35 ms, NEX = 1, for the oval squid, TR/TE = 3000/48 ms, NEX = 2, for the cuttlefish and TR/TE = 3500/35 ms, NEX = 2, for the giant squid). The stacks of MRI data were then processed to reconstruct the three-dimensional structure of each optic lobe using AVIZO software (FEI Company, USA).

### 2.4. Measurement and analysis

The size ratios of the eyeballs to optic lobes in the three species of cephalopods were determined by direct volume measurements. From the three-dimensional reconstructions of the MRI data, the extent of the concaveness on the lateral side of the optic lobe was calculated. This was estimated by finding the depth of the concave and dividing this by the presumed width of the optic lobe without the dent. Using only the middle section of the MRI images, the thickness of the cortex was determined by averaging 20 thickness measurements along the cortex and normalizing the result against its circumference. Similarly, the intensity of the inner granular layer was calculated by averaging 20 intensity measurements along the cortex and normalizing the result against the overall intensity of the optic lobe. These measurements were carried out using ImageJ software (National Institutes of Health, USA). All statistical analyses were done using one-way ANOVA, which was followed by the Tukey's test (Sigma Plot, USA).

## 3. Results

**Figure 2.**
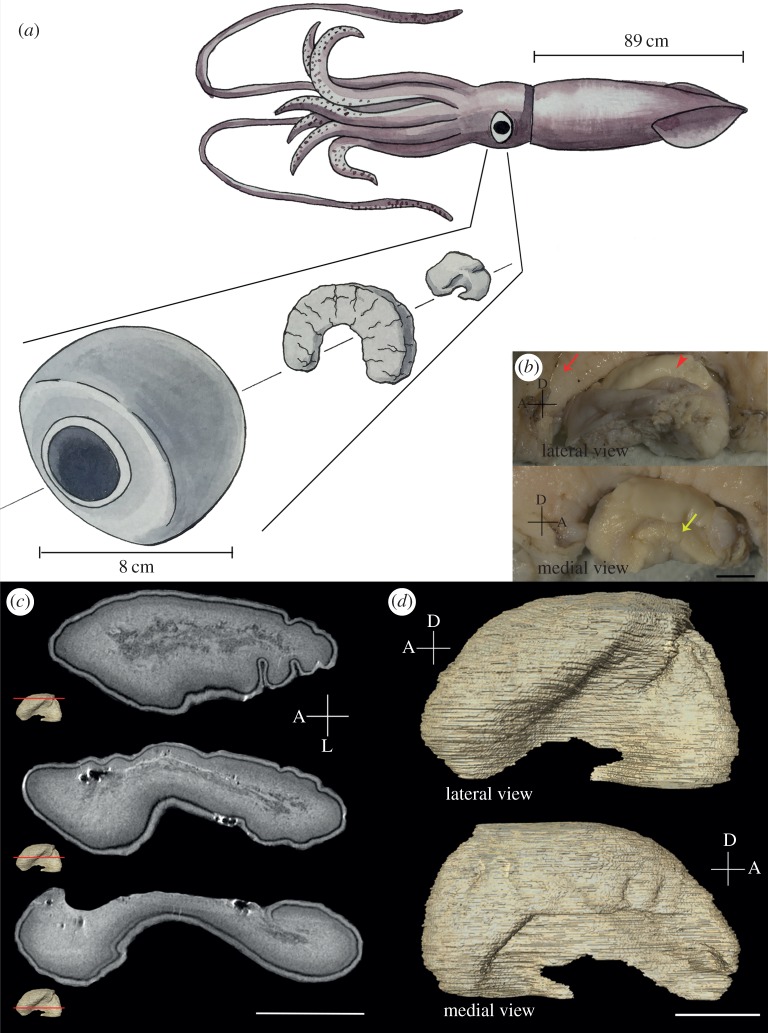
The optic lobe of *Architeuthis* is relatively flat and concave ventrolaterally with a prominent white body that surrounds dorsolaterally. (*a*) A schematic drawing of the optic lobe, white body and eyeball relative to its head. (*b*) The photos of dissected optic lobe (arrow head) and white body (arrow). The eyeball was removed to reveal optic nerve bundles and concave structure at the ventrolateral side. The medial view shows the optic tract region (arrow). Scale bar, 1 cm. (*c*) Three slices of images from the MRI scan showing the internal structure of the optic lobe. The bright region shows the neuropil-rich zone and the dark region represents the aggregation of cell somata. The cortex can be readily distinguished from the medulla in the MRI scan (see electronic supplementary material, Movie S1). The ventral region of the optic lobe is much narrower than the dorsal region, which confirms a significant concaveness on the ventrolateral side. Scale bar, 1 cm. (*d*) A three-dimensional rendition of the MRI scan showing the shape of the optic lobe in both lateral and medial views (see electronic supplementary material, Movie S2). D, dorsal; A, anterior; L, lateral. Scale bar, 1 cm.

Unlike typical squids, giant squids have a relatively small pair of optic lobes just behind their huge eyeballs; furthermore, there is an enlarged semi-annular tissue called the white body [21,22] that separates the eyeball and the optic lobe (figure 2*a*). The white body, a haematopoietic organ in cephalopods [23], is in contact with the dorsolateral side of the optic lobe and holds the eyeball in place. Numerous optic nerve fibres project from the retina directly into the lateral side of the optic lobe without passing through the white body (figure 2*b*, lateral view). The optic tract region, which is the main output site of the optic lobe, can be clearly seen on the medial side (figure 2*b*, medial view). The MRI scan of the optic lobe revealed for the first time that the giant squid has a relatively thicker cortex together with a less organized medulla (figure 2*c*; see also electronic supplementary material, Movie S1). Interestingly, the three-dimensional reconstruction of the optic lobe from the MRI scan showed that there is a prominent concave area on the ventrolateral side (figure 2*d*; see also electronic supplementary material, Movie S2).

**Figure 3.**
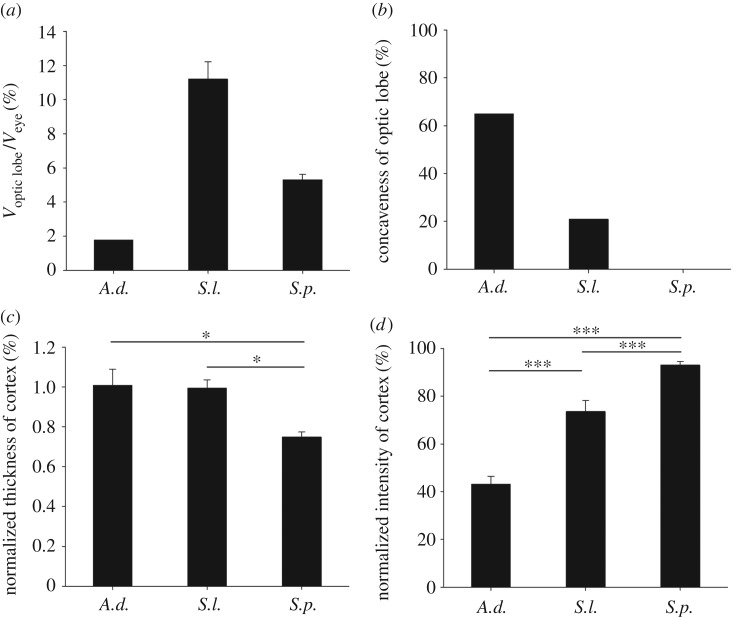
The optic lobe of *Architeuthis* is much smaller but contains more visual processing units than other cephalopods. (*a*) *Architeuthis* has a much smaller optic lobe relative to its eyeball when compared with other cephalopod species. (*b*) The lateral side of the optic lobe in *Architeuthis* is much more concave than that in other cephalopods. The percentage of concaveness was determined by calculating the depth of the dent area relative to the width of the optic lobe along the medial–lateral axis. (*c*) The cortical layer of the optic lobe in *Architeuthis* is relatively thicker than that of the cuttlefish. The thickness of the cortex was normalized against the circumference of the optic lobe. (*d*) The cortical layer of the optic lobe in *Architeuthis* is much denser than that of the other cephalopods. The cell density of the cortex was estimated by calculating the average intensity of the cortical layer, which has been normalized against the intensity of the whole optic lobe. A lower intensity indicates a higher cell density in the MRI scan. *A.d.*, *Architeuthis dux*; *S.l.*, *Sepioteuthis lessoniana*; *S.p*., *Sepia pharaonis*.

These morphological features in the optic lobe of the giant squid are more pronounced when compared with the two shallow-water cephalopod species, oval squid *Sepioteuthis lessoniana* and cuttlefish *Sepia pharaonis*, whose dynamic body patterning for communication and camouflage is sophisticated [24]. For example, it was apparent that the volume ratio of the optic lobe to the eye in the giant squid is much smaller than that in these two species (figure 3*a*). Similarly, the concaveness of the optic lobe in the giant squid is also much greater than that in these two species (figure 3*b*). Further quantification showed that the normalized thickness of the cortex is significantly greater in the giant squid than in cuttlefish (figure 3*c*). By contrast, the normalized intensity of the granular layer in the cortex is significantly lower in the giant squid than in other two species (figure 3*d*). The lower intensity of the granular layer of the optic lobe in the MRI scan indicates a higher density of cell somata; this observation suggests that the giant squid has relatively more cortical cells in the optic lobe available for the processing of visual information from the eye.

## 4. Discussion

Living in deep sea with a pair of enormous eyeballs as part of its head, the brain of the giant squid is subjected to significant compression due to the two eyes. It has been reported that the white body is a haematopoietic organ, namely a site for haematocyte formation in coleoid cephalopods [21,23]. Although not related to its original function, in the giant squid, the unusually large white body surrounds the optic lobe dorsolaterally (figure 2*a*) and is likely to function as a cushion for the huge eyeball as well as a means of protecting the optic lobe [22]. As a consequence of the presence of the eyeball compression, the concaveness found on the ventrolateral side of the optic lobe in *Architeuthis* is much more prominent than in *Sepioteuthis* and *Sepia* (figure 3*b*). This is also consistent with the neural anatomy of Teuthida, where the distance of two eyes is closer than in Sepiida species [25], thus pressing the optic lobe laterally even more.

In addition to the highly compressed optic lobe in giant squids, there is a large mismatch in size between the eyeball and the optic lobe when these are compared with the other two cephalopod species (figure 3*a*). A previous anatomical study has also indicated that *Architeuthis* has the smallest ratio of the optic lobe to the rest of the whole brain among all cephalopods that have been examined [26]. This evidence suggests that the optic lobe is relatively less important in giant squids when compared with other lobes found in cephalopod brains. However, if the relative thickness of the cortex in the optic lobe of giant squids is compared with that of oval squids and cuttlefish, it was clear that the giant squid is not that different from oval squids, while being significantly thicker than in cuttlefish (figure 3*c*). This observation is largely consistent with an early histological study of the optic lobe of *Architeuthis* [22]. Furthermore, the fact that the intensity of the cortex in the giant squid is the lowest one among these three cephalopods (figure 3*d*) also suggests that the giant squid has relatively more cells in the cortex than the two shallow-water species of cephalopods examined. This implies that the size mismatch between the giant eye and the optic lobe in *Architeuthis* is likely to be a result of a much reduced medulla within the optic lobe.

The medulla of the optic lobe in cephalopods is a higher visuomotor centre that mainly controls the body patterns associated with various visual behaviours including camouflage and communication [18,27,28]. The finding that the giant squid has a relatively small medulla suggests that body pattern control is less complex in the Oegopsina, namely the giant squids and Humboldt squids, which are known to show simple flashing and flickering on their skins only [29]. This study thus provides the first evidence to support that giant squids with large eyes have well-developed visual processing systems within the optic lobe. Furthermore, these squids exhibit less demanding body patterning behaviours and have less well-developed visual communication and this is perhaps because they are deep-sea animals living in dim light from bioluminescence for most of their life cycle. The end result of this would seem to be an evolutionary drive towards disproportionally smaller optic lobes.

## Data accessibility

This work is an anatomical study, and the MRI data presented in this manuscript are shown in figures and the electronic supplementary material. The measurement data of the optic lobes are available from the Dryad Digital Repository: http://dx.doi.org/10.5061/dryad.n49p1 [30].
